# Proximal muscle weakness as the sole manifestation of Cushing’s disease, misdiagnosed as dermatomyositis: a case report

**DOI:** 10.1186/s13256-022-03649-4

**Published:** 2022-12-22

**Authors:** Marjan Jeddi, Mesbah Shams

**Affiliations:** Endocrinology and Metabolism Research Center, Shiraz University of Medical Sciences, 71345-1414, Shiraz, Iran

**Keywords:** Cushing, Proximal myopathy, Dermatomyositis, Case report

## Abstract

**Background:**

Cushing’s syndrome consists of signs and symptoms related to prolonged exposure to high levels of glucocorticoid, and should be considered in individuals with the discriminatory signs and symptoms. Proximal myopathy is an important discriminatory sign.

**Case presentation:**

We report the case of a 36-year-old Iranian man who presented with proximal muscle weakness. He visited a rheumatologist in an outpatient clinic, and according to proximal muscle weakness and heliotrope rash (based on the rheumatologist’s notes) with the impression of dermatomyositis, prednisolone and azathioprine were prescribed for him that did not improve his clinical status and he was gradually wheelchair dependent. He was admitted to the hospital for evaluation of paraneoplastic syndromes. Standard laboratory tests and imaging were unremarkable, other than a brain magnetic resonance imaging that demonstrated a 30 × 12 mm homogeneously enhancing mass in the sellar region with extension to the suprasellar area. He had serum cortisol of 295 ng/mL, and adrenocorticotropic hormone of 222 pg/mL (on 5 mg prednisolone twice daily), with a diagnosis of Cushing’s disease. He underwent two sessions of trans-sphenoidal surgery 4 months apart. After the first surgery, the proximal muscle weakness improved dramatically and he was walking with the aid of a walker, and after the second surgery he is walking without any aids.

**Conclusion:**

This case report emphasizes the high diagnostic importance of proximal muscle weakness as the sole presenting manifestation of Cushing’s syndrome/disease.

## Introduction

Cushing’s syndrome consists of signs and symptoms related to prolonged exposure to high levels of glucocorticoid. This syndrome may be the result of endogenous or exogenous hypercortisolism [1]. Exogenous hypercortisolism is mostly the result of glucocorticoid use for treatment of other disorders. Endogenous Cushing’s syndrome comprises two forms: adrenocorticotropic hormone (ACTH) dependent or ACTH independent; ACTH-producing pituitary adenoma and ectopic ACTH secretion by neoplasms cause ACTH-dependent Cushing’s syndrome, and adrenal hyperplasia, adenoma, and carcinoma are responsible for ACTH-independent Cushing’s syndrome [2].

The prevalence of the disease is highly variable, and pituitary-mediated ACTH production accounts for up to 80% of cases of Cushing’s syndrome [3].

The clinical phenotypes include weight gain, fatigue, weakness, delayed wound healing, easy bruising, bone pain, depression, mood swings, emotional reactivity, characteristic moon face, thin arms and legs, acne, hirsutism, proximal muscle weakness of shoulder and hip girdle muscles, paper-thin skin, and wide vertical purplish abdominal striae. Many of these features are common in the general population, therefore Cushing’s syndrome should be considered in individuals with the most discriminatory signs and symptoms, and proximal myopathy is one of these discriminatory signs [4]. Muscle weakness is reported by 40–70% of patients with known Cushing’s syndrome, and it appears to be more pronounced in females, with the most commonly involved muscles being the proximal muscles of lower extremities [5].

Here we describe a young man presenting with proximal muscle weakness that appeared afterwards to have ACTH-producing pituitary adenoma; highlighting the high discriminatory value of this symptom for evaluation of Cushing’s syndrome.

## Case presentation

A 36-year-old Iranian man, married, and resident in Shiraz, Fars Province in the south of Iran, presented to our institute with the chief complaint of proximal upper and lower extremities muscle weakness. His problem started 5 months ago when he noticed a lack of strength of his proximal muscles, often causing difficulties climbing stairs, standing, or walking. He did not complain of any weight change, sleep or mood disorder, headache, or visual changes. There was no surgical history, and no family history for endocrine or rheumatologic disorders. The patient is a water pipe smoker.

He visited an outpatient clinic and, according to his proximal myopathy and heliotrope rash (based on the rheumatologist note) with the impression of dermatomyositis, prednisolone and azathioprine were prescribed, which did not improve his symptoms and, owing to gradual progression of myopathy, he was wheelchair dependent.

**Fig. 1:**
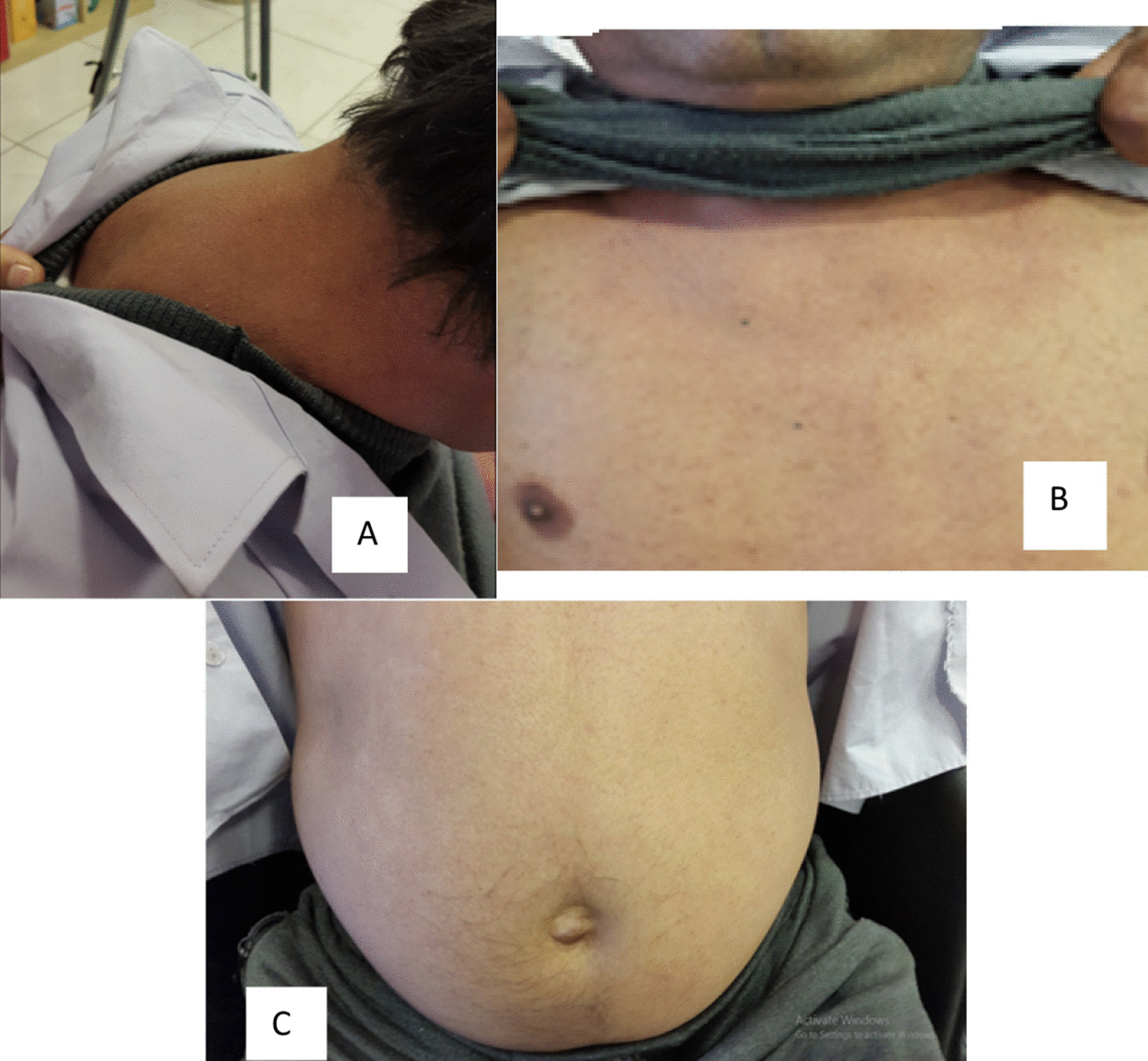
No buffalo hump (**A**), No shawl sign (**B**), No abdominal striae (**C**)

Four months later, he was referred by his physician for hospital admission and evaluation. On admission, he had a pulse rate of 92 beats per minute and blood pressure of 145/90 mmHg. His physical examination was normal, except a decrease in muscle power and deep tendon reflexes (muscle power was 4/5 in shoulders and arms, 3/5 in thigh muscles, biceps and triceps deep tendon reflexes were 1/2, and patella deep tendon reflex was 0/2). He failed to rise from squatting position and from sitting with arms crossed. His physical examination did not show any evidence of moon face, purple striae on the abdomen, easy bruising, buffalo hump, or visual abnormality (Fig. 1).

The patient was admitted to the rheumatology department. Initial workup showed normal muscle enzymes. Full serology of the patient is presented in Table 1.

**Table 1:** Serology at admission

Test	Value	Reference range
WBC (/L)	12.6 × 10^9^	4.00–11.0 × 10^9^
Hemoglobin (g/dL)	12.8	13–18
MCV (fL)	103.3	87 ± 7
Platelets	207,000	150,000–450,000
Total protein (g/dL)	5.6	6–8.3
Albumin (g/dL)	3.2	3.5–5.5
AST (units/L)	15	5–40
ALT (units/L)	17	7–56
Alkaline phosphatase (IU/L)	131	20 to 140
Glucose	99	72–108
CPK (IU/L)	25	39–308
LDH (IU/L)	658	140–280
ESR (mm/hour)	22	0–15
CRP (mg/dL)	3	< 10
BUN (mg/dL)	12	7–20
Creatinine (mg/dL)	0.9	0.6–1.2
Na (mEq/L)	140	135–145
K (mEq/L)	3.6	3.5–5.0
Calcium (mg/dL)	7.6	8.5–10.2
Phosphate (mg/dL)	2.8	2.5–4.5
C3 (g/L)	1.37	0.9–1.8
C4 (g/L)	0.2	0.1–0.4
ANA	0.17	Negative < 0.8
Anti-dsDNA	6.6	Negative < 16
Rheumatoid factor (RF) (IU/mL)	13	Up to 14
cANCA	< 2.0	Normal < 10
pANCA	< 2.0	Normal < 10
SSA-RO	2	0–5
SSB-LA	2	0–5
Anti-Sm	2	0–5
Anti-Jo1	3	0–5
Anti SCL-70	2	0–5
Anti-RNP/Sm	2	0–5
Anti-mitochondrial Ab	0.1	< 12

*WBC* White blood cells,* MCV* mean corpuscular volume,* AST* aspartate aminotransferase,* ALT* alanine transaminase,* CPK* creatine phosphokinase,* LDH* lactate dehydrogenase,* ESR* erythrocyte sedimentation rate,* CRP* c-reactive protein,* BUN* blood urea nitrogen,* Na* sodium,* K* potassium, *C3* complement,* C4* complement,* ANA* antinuclear antibody,* Anti ds DNA* double stranded DNA antibody,* C-ANCA* antineutrophil cytoplasmic antibody,* P-ANCA* perinuclear anti-neutrophil cytoplasmic antibody,* SSA-RO* anti–sjögren's-syndrome-related antigen A autoantibody,* SSB-LA* anti–Sjögren’s-Syndrome-related antigen B autoantibody,* Anti Sm* autoantibodies to smith,* Anti jo1* an anti-nuclear antibody,* Anti SCL-70* anti-topoisomerase antibody seen mainly in diffuse systemic scleroderma,* Anti RNP/Sm* autoantibodies directed against the small nuclear ribonucleoprotein

Electromyography (EMG) and nerve conduction velocity (NCV) revealed a decreased velocity in muscle fiber conduction, indicative of a neuromyopathic process, and recommended that paraneoplastic syndromes should be considered. For locating primary malignancy, some imaging studies were performed including chest X-ray, abdomen–pelvic sonography, brain computed tomography (CT) scan, and lumbosacral magnetic resonance imaging (MRI). Abdomen–pelvic sonography findings were unremarkable; Lumbosacral MRI showed decreased vertebral body height with enhancement noted in multiple lumbar vertebral bodies (especially in L3), suggestive of multilevel pathologic fracture, and atrophic change of paraspinal muscles. Brain CT scan showed a round, well-defined hyperdense structure measuring 14 × 13 mm in the sellar and suprasellar areas, with the possibility of anterior communicating artery aneurysmal dilation or sellar mass.

**Fig. 2:**
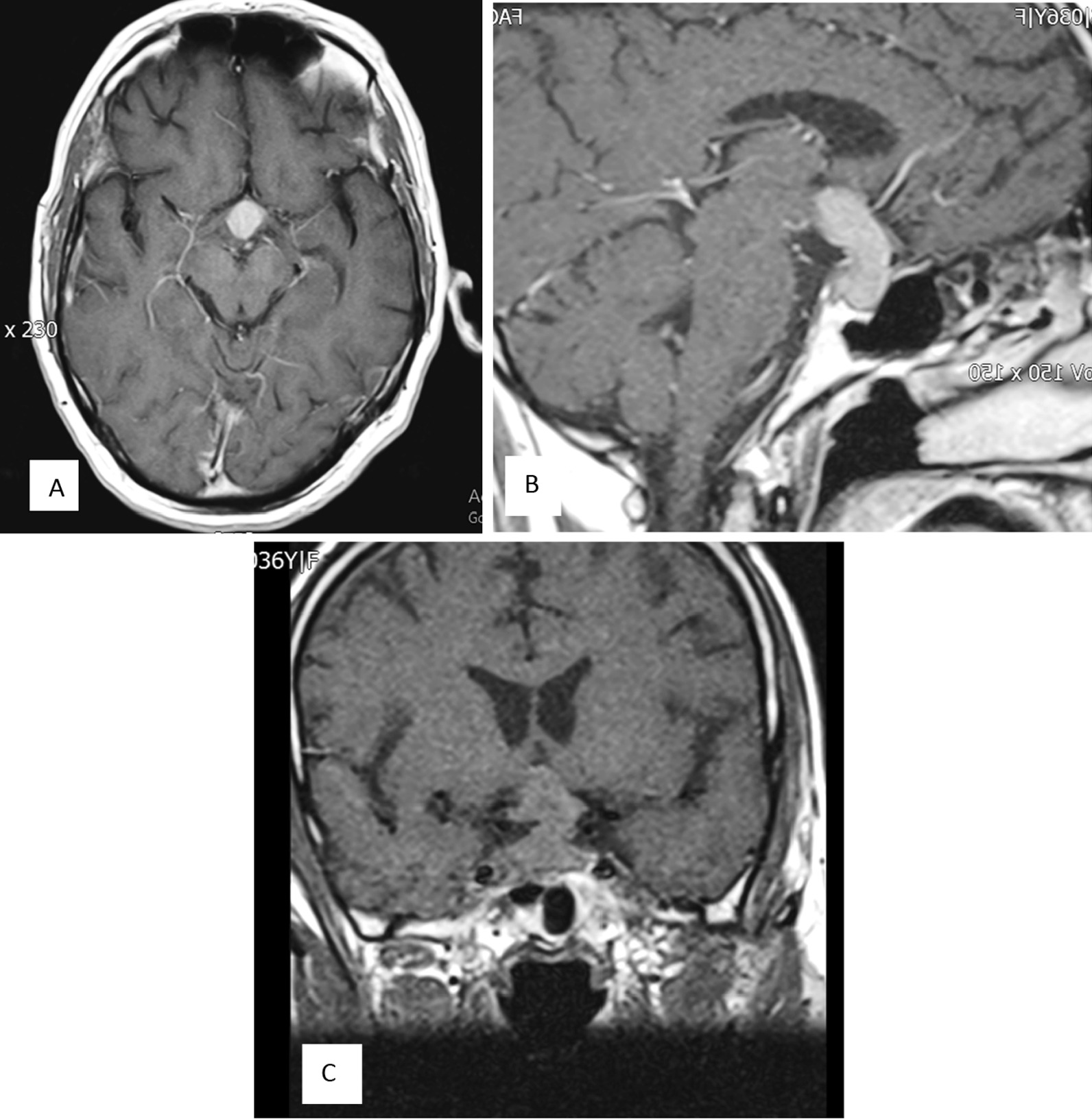
A 30 × 12 mm homogeneously enhancing mass in the sellar region with extension to the suprasellar region, causing pressure effect on the optic chiasma and invasion to the right side cavernous sinus (surrounding 50% of cavernous portion of right carotid artery), and involvement of posterior clinoid processes. Axial view (**A**). Sagittal view (**B**). Coronal view (**C**)

Brain MRI demonstrated a 30 × 12 mm homogeneously enhancing mass in the sellar region with extension to the suprasellar region and a pressure effect on the optic chiasma and invasion to the right side of the cavernous sinus and involvement of posterior clinoid processes, suggestive of pituitary macroadenoma versus meningioma (Fig. 2).

Table 2 demonstrates the endocrine workup of the patient (Table 2).

**Table 2:** Biochemical hormone assay of the patient

Test	Value	Reference range
Prolactin (ng/mL)	24.9	3.46–19.4
IGF-I (ng/mL)	79	68–220
Growth hormone (ng/mL) fasting	0.14	0.4–10
Growth hormone (ng/mL) 60 minutes after 75 g oral glucose	0.03	< 0.5
Growth hormone (ng/mL) 120 minutes after 75 g oral glucose	0.06	< 0.5
LH (mIU/mL)	0.48	0.57–12.07
FSH (IU/L)	0.85	0.95–11.95
Testosterone (ng/dL)	44.0	227–1030
Free testosterone (pg/mL)	4.87	9.1–32.2
T3 (nmol/L)	0.85	0.89–2.44
T4 (µg/dL)	3.56	4.87–11.72
TSH (µIU/mL)	1.03	0.27–5.95
Cortisol, 8.00 AM (ng/mL) (on prednisolone 5 mg twice daily)	295.0	54.9–287.5
ACTH (pg/ml) (on prednisolone 5 mg twice daily)	222	Up to 46

*IGF-I* insulin-like growth factor 1,* LH* luteinizing hormone,* FSH* follicle stimulating, hormone,* TSH* thyroid-stimulating hormone,* ACTH* adrenocorticotropic hormone

Following the diagnosis of Cushing’s disease, trans-sphenoidal surgery was scheduled for the patient. Pathologic report of pituitary lesion was signed out as pituitary adenoma.

In the follow-up visit, 2 months after trans-sphenoidal surgery (TSS), the patient’s condition had partially improved. He was on levothyroxine 100 µg daily, his muscle weakness improved, and he can walk using a walker; his proximal muscle power was 4/5 in lower extremities.

At this time, follow-up MRI was performed, which showed a 28 × 14 × 13.4 mm sellar mass, isointense in T2W1 and hypersignal in T1W1 with extension to the suprasellar region. We did not find suppression of cortisol after low-dose (4 mg) dexamethasone (cortisol: 283.9 ng/mL; normal (NL): 54.9–287.5 ng/mL). The endocrinology and neurosurgery teams of our institute decided to reoperate. The patient underwent further surgery 6 months after the first operation, and now, 1 year after the second operation, he is not wheelchair bound, can walk without help, and uses levothyroxine 100 µg daily, testosterone 250 mg monthly, alendronate 70 mg weekly, and calcium and vitamin D supplements.

## Discussion

Our patient presented with proximal myopathy, vertebral pathologic fracture, and inability to walk was first diagnosed with dermatomyositis and treated with glucocorticoid. It took approximately 6 months to identify ACTH-producing pituitary adenoma as responsible for his symptoms.

The differential diagnosis of proximal myopathy includes an acquired or genetic myopathic disorder [6]. The acquired causes may be infectious, neurologic, endocrine, inflammatory, rheumatologic, metabolic, electrolyte induced, or drug induced [7].

Chronic glucocorticoid excess directly causes muscle weakness by binding to steroid hormone receptors on the skeletal muscles; this hormone can change muscle lipids, carbohydrates, and protein metabolism [8]. The main effect of glucocorticoids is catabolism of muscle proteins via the fork head box O3 (*FOXO3*) pathway, intramuscular fat accumulation, and atrophy of type 2 muscle fibers [9]. In addition, a high level of ACTH may be directly myopathic, regardless of glucocorticoid synthesis [8]. It seems that the effect of biochemical remission of Cushing’s syndrome on myopathy-related fatigability in long-term follow-up is not satisfactory [10], ranging from limited improvement to persistence despite remission of hypercortisolism [10, 11]. Age, waist-to-hip-ratio, and a hyperglycemic metabolic state are the main risk factors for myopathy, and intact postoperative growth hormone and insulin-like growth factor (IGF) is essential for its restoration [9].

Cushing’s syndrome is an important cause of secondary osteoporosis and fragility fractures. The effects of glucocorticoid on decreasing number of osteoblasts, inhibition in secretion of gonadotropins and growth factors, and reducing calcium absorption results in bone loss, which is more prominent on trabecular bones than cortical bones. Low bone mineral density in children or young women and vertebral fractures may be the presenting features of Cushing’s syndrome [12]. Osteoporosis occurs in 50% of patients with Cushing’s syndrome [13], more frequently in ACTH-independent Cushing’s syndrome than in Cushing’s disease, and causes fracture in 30–50% of individuals [14]. After treatment of Cushing’s syndrome, improvement in osteoblast function results in recovery in bone density within 12–36 months after the level of cortisol is normalized [13].

Dermatomyositis is a multisystem disorder defined by autoimmune myopathic involvement of striated muscle and proximal and symmetric muscle weakness is the most common feature of this idiopathic inflammatory disease [15]. The association between dermatomyositis and malignancy, considered as paraneoplastic syndrome, is well known. Dermatomyositis accompanied with malignancy may present before, concurrently with, or after diagnosis of cancer [16]. Therefore, early manifestations of dermatomyositis should precipitate the physician to evaluate the patient for suspicious malignancy.

Regarding clinical findings and disease progression in our patient, three probable diagnoses should be considered: one impression is dermatomyositis as a paraneoplastic syndrome, as a sign of indolent cancer. On screening for malignancy, we did not find any positive result and the patient’s disability improved after pituitary surgery. The other impression could be iatrogenic glucocorticoid-induced Cushing’s syndrome; however, our patient had a high level of ACTH while using steroids, and therefore this possibility is very unlikely. The third probable impression is Cushing’s syndrome as the cause of proximal myopathy and the contradictory diagnosis of dermatomyositis as the first diagnosis. We did not find characteristic cutaneous manifestation of dermatomyositis and myositis-associated antibody in this patient; we think he is a case of ACTH-dependent Cushing’s syndrome, presenting solely with proximal muscle weakness and vertebral fracture. After trans-sphenoidal surgery, his myopathy improved.

O’Leary and Waisman reported 40 cases of dermatomyositis seen at the Mayo Clinic, of which two patients had features of Cushing’s syndrome. It seems that muscle weakness and microscopic features in the affected muscles of Cushing’s syndrome can mimic dermatomyositis [17].

Minou Tran reported a 41-year-old man with progressive muscle weakness to the point that he was bedridden. After diagnosis of Cushing’s disease from biochemical data and an intrasellar pituitary mass measuring 1.0 × 1.5 cm, he underwent trans-sphenoidal resection of the presumed pituitary ACTH-producing macroadenoma. Postoperatively he had a high level of ACTH and cortisol supporting the residual tumor. The patient was complicated, with progressive clinical deterioration, and a second surgery was postponed and unfortunately he died after 3 weeks [18].

Vilim Molnar reported a 35-year-old woman presenting with a 1-year history of foot pain without any history of trauma. After detection of multiple metatarsal fractures by radiography, and evaluation for secondary causes of osteoporosis, the diagnosis of Cushing’s disease was confirmed, although other typical signs and symptoms were not remarkable [19].

## Conclusion

Muscle weakness is one of the frequent symptoms and a relevant comorbidity reported in Cushing’s syndrome [5]. Our patient, who had a 6-month history of difficulties climbing stairs and walking and needing a wheelchair, was misdiagnosed and treated as a case of dermatomyositis. After confirmation of Cushing’s disease and surgery, his muscle weakness improved dramatically and he is now walking without any aid. This case report emphasizes the high diagnostic value of proximal muscle weakness, which can be the sole presenting manifestation of Cushing’s syndrome/disease, without any other typical signs or symptoms.

## Data Availability

The datasets are available from the corresponding author on reasonable request.
